# Association between sensitivity to thyroid hormone indices and frailty in the elderly: a cross-sectional study

**DOI:** 10.3389/fendo.2025.1463283

**Published:** 2025-07-03

**Authors:** Xinyu Qi, Jia Fang, Jiru Zhang, Jihai Chen, Daowen Zhang, Runqing Shi, Yusheng Zhang, Lijun Wei, Lu Yan, Yunlu Sheng, Guoxian Ding, Xiaojun Ouyang, Yu Duan

**Affiliations:** ^1^ Department of Geriatric Endocrinology, The First Affiliated Hospital of Nanjing Medical University, Nanjing, China; ^2^ Department of Geriatric, Geriatric Hospital of Nanjing Medical University, Nanjing, China

**Keywords:** aging, thyroid function, thyroid hormone sensitivity indicators, frailty, sarcopenia

## Abstract

**Objective:**

Frailty is a prevalent geriatric syndrome that affects several systems and lacks accurate evaluation indications. Previous studies on the association between thyroid function and frailty have contradicted. This study introduced the thyroid hormone sensitivity indices to analyze their connection with frailty and evaluate its usefulness in assessing frailty.

**Methods:**

A total of 1030 Chinese elderly individuals were included. The FRAIL scale was utilized to assess the frailty status of subjects. The thyroid hormone sensitivity indices, including the following: Thyrotrophic Thyroxine Resistance Index (TT4RI), Thyroid Stimulating Hormone Index (TSHI), Thyroid Feedback Quantile-based Index (TFQI) and Free Triiodothyronine/Free Thyroxine (FT3/FT4), were calculated based on thyroid function. A multivariate logistic regression analysis assessed the association between frailty and these markers.

**Results:**

The study findings indicated that frailty groups exhibited lower levels of FT3 and TSH compared to non-frailty groups. Additionally, the central sensitivity index of thyroid hormone [TFQI(FT3)] and the peripheral sensitivity index (FT3/FT4) were also lower in frailty groups. Following the adjustment for multiple variables, FT3 (OR=0.640, 95%CI: 0.534-0.768, *p <*0.001), TSHI (OR=0.829, 95%CI: 0.708-0.971, *p* =0.020), TFQI(FT3) (OR=0.358, 95%CI: 0.233-0.550, *p <*0.001) and FT3/FT4 (OR=0.006, 95%CI: 0.001-0.058, *p <*0.001) showed a negative connection with the score of FRAIL scale.

**Conclusion:**

Serum FT3 and TSH declined in frail patients, whereas there was no alteration in serum FT4. Both the decrease of central sensitivity index of thyroid hormone, TSHI and TFQI(FT3), and peripheral sensitivity index, FT3/FT4, were associated with frailty assessed by FRAIL scale. Specific thyroid measures, especially FT3, TFQI(FT3) and FT3/FT4, would be served as easily measurable indications that are essential for evaluating frailty.

## 1 Introduction

Frailty is a geriatric syndrome that affects multiple systems and is frequently observed in older individuals. It is characterized as a non-specific clinical condition with a decrease in biological reserve capacity, significantly increased vulnerability and functional decline against stress, which is caused by many physiological, physical, mental and other functional impairments (1). The process of frailty gradually and consistently emerges as individuals grow older. People who show early indicators of frailty typically display hidden and vague symptoms. As the condition advances, even small external stimuli or physical stressors could lead to negative consequences such as disability, protracted illness, depression, cognitive decline, and death (2). Early intervention in geriatric populations holds critical clinical significance, as the pre-frailty phase may represent a reversible window for restoring functional resilience. Current frailty assessment protocols predominantly rely on phenotypic screening scales, while lacking validated biomarkers to objectively quantify subclinical deterioration and guide targeted therapeutic strategies (3). Therefore, there is still a shortage of sensitive indicators for supplementary evaluation of frailty.

Sarcopenia is a core component of frailty, manifested as age-related declines in skeletal muscle mass, strength or function (4). Our previous study on muscle condition and thyroid function in older individuals revealed a negative correlation between the levels of free triiodothyronine (FT3) and total triiodothyronine (TT3) with sarcopenia. This study demonstrated that thyroid hormone (TH), particularly triiodothyronine (T3), could serve as a dependable indicator for sarcopenia (5).

Thyroid hormone is a key factor in growth and energy metabolism, and changes in thyroid function were believed to be closely related to the occurrence of frailty (6). However, depending simply on TH may not provide a comprehensive and accurate representation of an individual thyroid function. This study has introduced the thyroid hormone sensitivity index to investigate variations in TH levels among frail individuals and assess the relationship between the thyroid hormone sensitivity index and senile frailty, aiming to evaluate the usefulness of each thyroid hormone sensitivity index component in assessing frailty.

## 2 Methods

### 2.1 Study subjects

A total of 1884 individuals were included in this cross-sectional study, all receiving a health checkup at the Geriatric Hospital of Nanjing Medical University from January 2016 to June 2023. During the initial recruitment phase, only individuals who fulfilled the following criteria were selected to participate in our study: 1) Possessing the capacity to complete physical examinations and questionnaires; 2) Capable of providing a concise and comprehensive medical background. The exclusion criteria were as follows: 1) Under the age of 60 years (n=301); 2) With missing key data (n=546); 3) Data exception (n=7), such as a patient with an FT3 = 3.08 pmol/L who had an FT4 test result of 1151.00 pmol/L, which contradicted logical expectations. Following the exclusion process, a grand number of 1030 individuals were ultimately incorporated. This study was approved by the Ethics Committee of the Geriatric Hospital of Nanjing Medical University. All subjects have signed informed consent.

### 2.2 Data collection

The participants were required to complete a self-assessment questionnaire, that included information about gender, age, weight, height, smoking and drinking habits, exercise routine, and medical history. Body Mass Index (BMI) was calculated by dividing weight (kg) by height square (m²). According to China’s BMI criteria, participants were classified as emaciation (BMI <18.5kg/m^2^), normal (18.5~23.9kg/m^2^), overweight (24~27.9kg/m^2^), and obesity (BMI ≥28kg/m^2^) (7). Participants were organized to fill in the Nutritional Risk Screening 2002 (NRS2002) and the Simple Mental State Scale (MMSE) to assess the nutritional status and cognitive level of the patients respectively. NRS2002 (8) consisted of three components: disease status, nutritional intake and age. If the NRS2002 score was ≥3, the subject was considered to be in a state of malnutrition. MMSE (9) with a total score of 30 was used to evaluate patients across seven aspects: time orientation, place orientation, immediate memory, attention and calculation, delay memory, language and visuospatial skills. If the MMSE score was under 24, the subject was judged to suffer from cognitive decline (10).

### 2.3 Thyroid function tests and thyroid hormone sensitivity index calculation

Thyroid function was detected by chemiluminescence immunoassay (Roche electroluminescence apparatus E170) at the clinical laboratory center of Geriatric Hospital of Nanjing Medical University. The reference ranges of the normal thyroid function values were as follows: FT3 2.43~6.01 pmol/L, FT4 9.01~19.05 pmol/L, TSH 0.35~4.94 mIU/L.

The thyroid hormone sensitivity index was assessed from the central and peripheral parts. FT3/FT4 ratio was on behalf of the TH peripheral sensitivity index (11). The higher the FT3/FT4 ratio was, the better the peripheral sensitivity to TH was. TH central sensitivity index was represented by the following three components: Thyrotrophic Thyroxine Resistance Index (TT4RI) (12), Thyroid Stimulating Hormone Index (TSHI) (13) and Thyroid Feedback Quantile-based Index (TFQI) (14). TSHI, TT4RI and TFQI elevation corresponded to a decrease in TH central sensitivity. TFQI was calculated using the cumulative distribution function (CDF) and ranged from -1 to 1. A TFQI of 0 showed normal TH central sensitivity. TFQI (FT3) was derived using TFQI(FT4) formula to investigate the impact of FT3 on the hypothalamus-pituitary-thyroid axis (HPT axis). The calculation formulas were as follows:


FT3/FT4=FT3(pmol/L)/FT4(pmol/L)

TT4RI=FT4(pmol/L)×TSH(mIU/L)

TSHI=ln TSH(mIU/L)+0.1345×FT4(pmol/L) 

TFQI=cdf FT4(pmol/L)−(1−cdf TSH(mIU/L))

### 2.4 Screening for frailty

The FRAIL scale (15), created by International Academy on Nutrition and Aging (I.A.N.A), was utilized to assess frailty in patients. This scale has demonstrated efficacy in predicting the likelihood of disability and mortality in older individuals. The frailty scale comprised five components: fatigue, resistance, ambulation, illnesses and weight loss. Subjects who met three or more items were diagnosed as frailty, those who met 1–2 items were pre-frailty, and those who did not meet them were considered as non-frailty.

### 2.5 Diagnosis of sarcopenia

Of all participants, subjects who were able to measure grip strength (three times with a hand dynamometer, recording the maximum value, Jamar^®^, Los Angeles, CA, USA), calculate a 6-meter pace (at least two times, averaging) and complete bioelectrical impedance analysis (BIA) (InBody S10) were further evaluated for sarcopenia. According to the definition and diagnostic criteria of sarcopenia in AWGS2019 (4), males with skeletal muscle index (SMI) <7.0kg/m^2^, average 6-meter pace <1m/s^2^ and/or maximum grip strength <28kg were diagnosed with sarcopenia while the female with SMI <5.7kg/m^2^, average 6-meter pace <1m/s^2^ and/or maximum grip strength <18kg were. SMI was calculated from appendicular skeletal muscle mass (ASM) measured by BIA, and the specific calculation formula was as follows:


$$\text{SMI}\left(\text{kg}/{\text{m}}^{2}\right)=\text{ASM}\left(\text{kg}\right)/\text{heigh}{\text{t}}^{2}\left({\text{m}}^{2}\right)$$

### 2.6 Statistical analysis

The data were analyzed using Stata MP 17 statistical software. The Kolmogorov-Smirnov test was employed to assess the normality of the data distribution. Continuous variables that followed a normal distribution were compared using a t-test or ANOVA. On the other hand, continuous variables that did not follow a normal distribution were compared using the Mann-Whitney U test or the Kruskal-Wallis H test. The comparison of categorical variables was conducted using the chi-square test. Data were expressed as means ± standard deviations or medians (interquartile ranges) for continuous variables and percentage (%) for categorical variables. The study employed ordered logistic regression analysis to examine the correlation between thyroid function and frailty as well as correlation between TH sensitivity indicators and frailty. All statistical tests were two-sided; *p*-values < 0.05 were considered statistically significant.

## 3 Results

### 3.1 Baseline data, TH and TH sensitivity for participants

A total of 1030 participants (including 605 males and 425 females) were enrolled in this cross-sectional study. Based on the FRAIL scale, individuals were categorized into three groups: non-frailty, pre-frailty and frailty (as shown in **Table 1**). The prevalence of frailty was found to be 19.7%. The individuals in pre-frailty group (with a median age of 86 years) and frailty group (with a median age of 88 years) were significantly older than those in non-frailty group (with a median age of 78 years) (*p <*0.001). The proportion of individuals in frailty group who had a history of drinking, lack of exercise, emaciation, malnutrition, coronary heart disease, stroke and cognitive decline was considerably higher compared to the non-frailty group (*p <*0.05). There was no significant variation in the occurrence of smoking (*p* =0.265), hypertension (*p* =0.112) and diabetes (*p* =0.827) among three groups.

**Table 1:** Characteristics of the elderly population (age ≥ 60) in different frailty states (n=1030).

Characteristics	Non-frailty (n=334)	Pre-frailty (n=493)	Frailty (n=203)	*p*-value
Age, years	78(71~86)	86(80~89)	88(84~92)	<0.001
Female, %	33.53	47.46	38.92	<0.001
Smoking, %	21.69	17.48	20.20	0.265
Drinking, %	34.53	20.33	19.31	<0.001
BMI				0.034
Emaciation, %	1.21	4.38	7.36	
Healthy, %	44.11	41.58	42.33	
Overweight, %	39.88	36.98	34.36	
Obesity, %	14.80	17.07	15.95	
Malnutrition, %	13.15	22.96	44.72	<0.001
Hypertension, %	65.87	69.78	74.38	0.112
CHD, %	30.84	34.08	42.36	0.023
DM, %	43.71	42.39	44.83	0.827
Stroke, %	35.93	51.72	61.58	<0.001
Sporting, %	89.56	60.39	36.19	<0.001
Cognitive Decline, %	18.02	35.38	50.25	<0.001

Data are expressed as means ± standard deviations or medians (interquartile ranges) for continuous variables and percent (%) for categorical variables. *P*-value is calculated by H-test for continuous variables, Chi-square test for categorical variables.

CHD, Coronary heart disease; DM, Diabetes mellitus.

**Figure 1:**
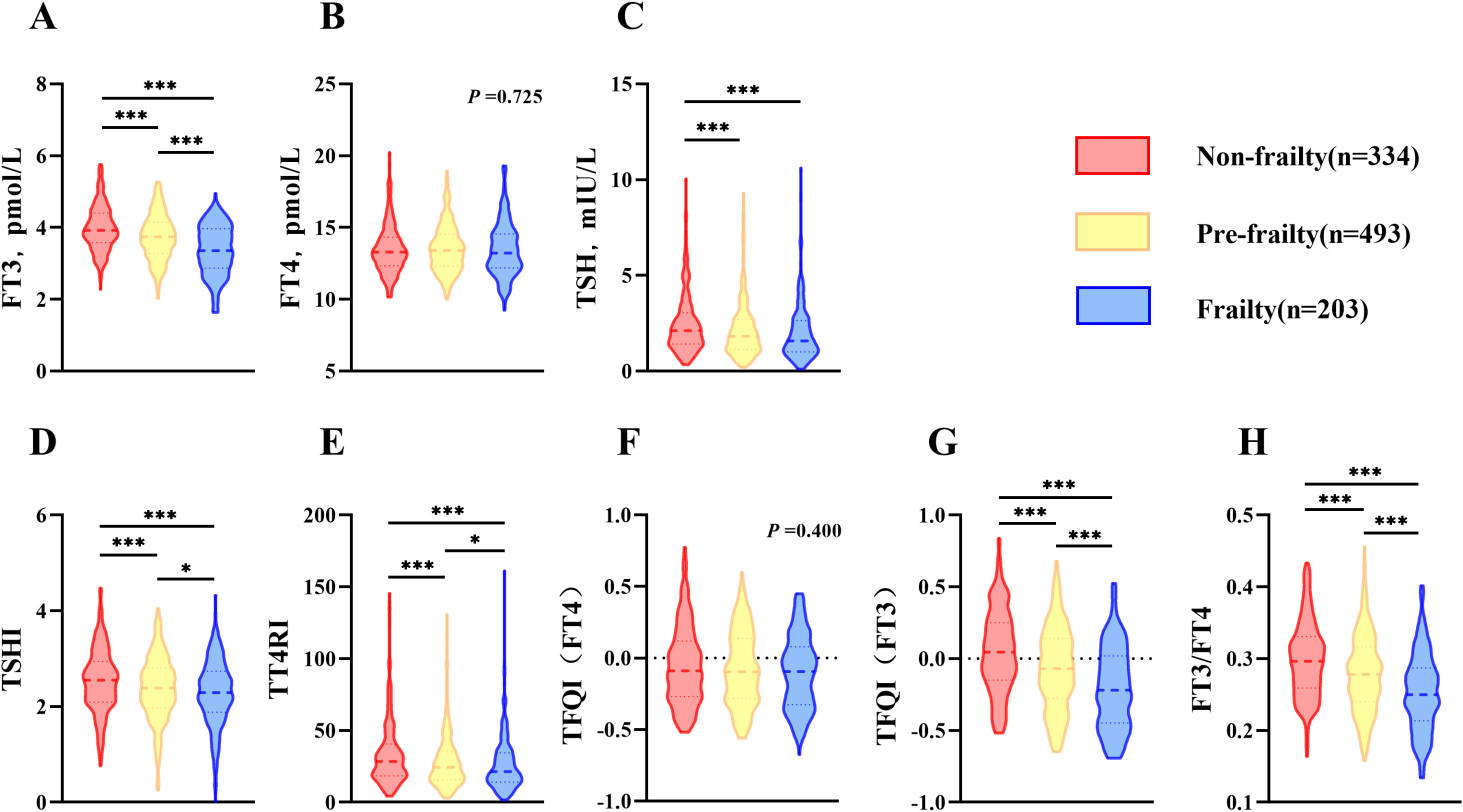
Comparison of thyroid function and thyroid hormone sensitivity index in elderly population (age≥60). **(A)** Comparison of FT3 among groups; **(B)** Comparison of FT4 among groups; **(C)** Comparison of TSH among groups; **(D)** Comparison of TSHI among groups; **(E)** Comparison of TT4RI among groups; **(F)** Comparison of TFQI(FT4) among groups; **(G)** Comparison of TFQI(FT3) among groups; **(H)** Comparison of FT3/FT4 among groups. H-test was used to compare and analyze the data differences among groups. If the difference among three groups was significant, further analysis was conducted to discern the differences between each pair of groups. The P-value represented the comparison result among three groups, along with the p-value for each pair of groups. *p <0.05, ***p <0.001. TSHI, Thyroid Stimulating Hormone Index; TT4RI, Thyrotrophic Thyroxine Resistance Index; TFQI, Thyroid Feedback Quantile-based Index; FT3/FT4, Free Triiodothyronine/Free thyroxine.

The thyroid function of each group was compared respectively (**Figure 1**). In frailty group, both FT3 and TSH were significantly lower than those in non-frailty group (*p <*0.001). There was no significant difference in FT4 among three groups (*p* =0.725).

The components of TH sensitivity index corresponding to each group were used for comparison respectively (**Figure 1**). Compared with non-frailty group, central sensitivity index of TH [TSHI, TT4RI and TFQI(FT3)] and peripheral sensitivity index of TH (FT3/FT4) decreased prominently in frailty group (*p <*0.001), while TFQI (FT4) exhibited no significant difference (*p* =0.400).

### 3.2 Association of thyroid function and TH sensitivity index with frailty between octogenarians and beyond

**Figure 2:**
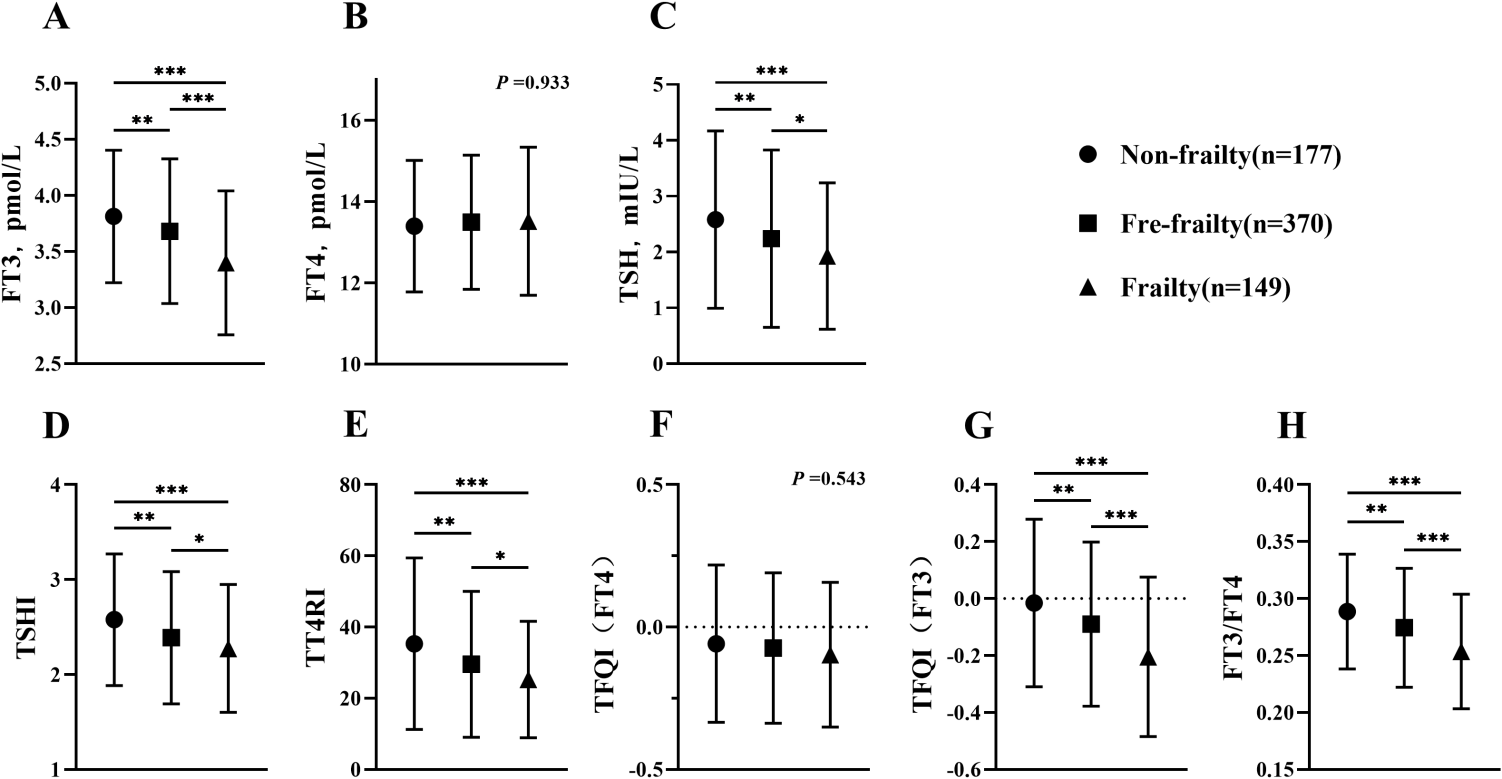
Comparison of thyroid function and thyroid hormone sensitivity index between octogenarians and beyond. **(A)** Comparison of FT3 among groups; **(B)** Comparison of FT4 among groups; **(C)** Comparison of TSH among groups; **(D)** Comparison of TSHI among groups; **(E)** Comparison of TT4RI among groups; **(F)** Comparison of TFQI(FT4) among groups; **(G)** Comparison of TFQI(FT3) among groups; **(H)** Comparison of FT3/FT4 among groups. H-test was used to compare and analyze the data differences among groups. If the difference among three groups was significant, further analysis was conducted to discern the differences between each pair of groups. The P-value represented the comparison result among three groups, along with the p-value for each pair of groups. *p <0.05, **p <0.01, ***p <0.001. TSHI, Thyroid Stimulating Hormone Index; TT4RI, Thyrotrophic Thyroxine Resistance Index; TFQI, Thyroid Feedback Quantile-based Index; FT3/FT4, Free Triiodothyronine/Free thyroxine.

To better observe the association of thyroid function and TH sensitivity index with age in the frail, 696 subjects aged ≥80 years were selected for subgroup analysis, in which the incidence of frailty was 25.4% (**Figure 2**). When comparing thyroid function in three groups, it was shown that levels of FT3 and TSH were reduced due to frailty (*p <*0.05), whereas levels of FT4 did not show significant differences (*p* =0.933). When analyzing the TH sensitivity index in three different groups, it was shown that TSHI, TT4RI, TFQI(FT3) and FT3/FT4 were considerably lower in frailty group compared to other groups (*p <*0.05). When comparing the oldest-old group to subjects aged below 80 and over 60, there was a significant decrease in FT3, TFQI (FT3) and FT3/FT4 in the oldest-old group (*p <*0.001). However, there was no significant difference in FT4, TSH, TSHI and TFQI(FT4) (*p* =0.5267, *p* =0.9637, *p* =0.9616, *p* =0.9849, respectively).

### 3.3 Association of thyroid function and TH sensitivity index with frailty in euthyroid subjects

**Figure 3:**
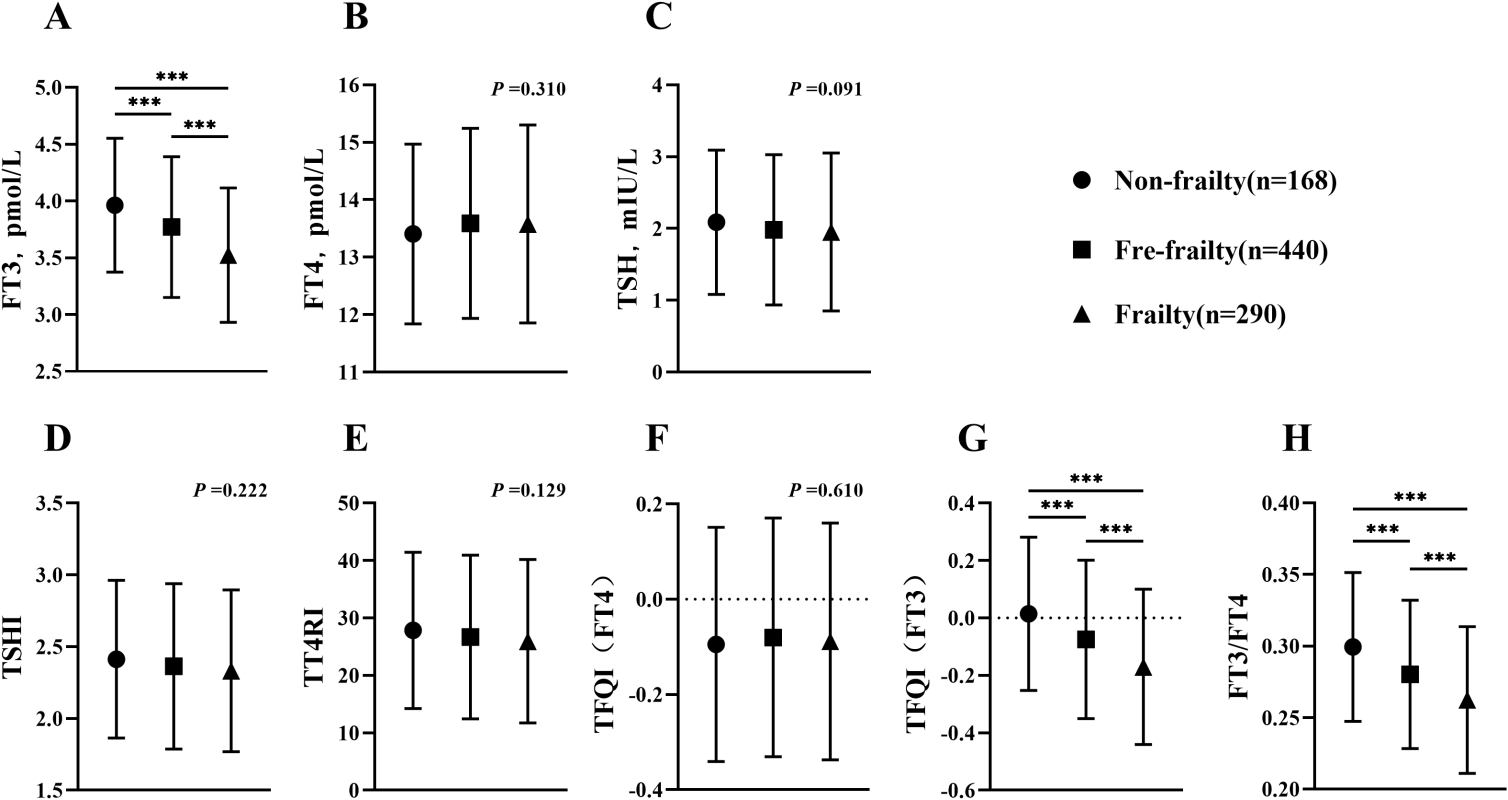
Comparison of thyroid function and thyroid hormone sensitivity index in euthyroid subjects (age≥60). **(A)** Comparison of FT3 among groups; **(B)** Comparison of FT4 among groups; **(C)** Comparison of TSH among groups; **(D)** Comparison of TSHI among groups; **(E)** Comparison of TT4RI among groups; **(F)** Comparison of TFQI(FT4) among groups; **(G)** Comparison of TFQI(FT3) among groups; **(H)** Comparison of FT3/FT4 among groups. H-test was used to compare and analyze the data differences among groups. If the difference among three groups was significant, further analysis was conducted to discern the differences between each pair of groups. The P-value represented the comparison result among three groups, along with the p-value for each pair of groups. ***p <0.001. TSHI, Thyroid Stimulating Hormone Index; TT4RI, Thyrotrophic Thyroxine Resistance Index; TFQI, Thyroid Feedback Quantile-based Index; FT3/FT4, Free Triiodothyronine/Free thyroxine.

In order to assess the influence of thyroid function on outcomes, a subgroup analysis was conducted on a sample of 898 individuals without thyroid dysfunction. Among this group, 18.7% were found to have frailty (**Figure 3**). When comparing thyroid function in three groups, it was found that the frailty group had a significant reduction in FT3 levels (*p <*0.001). Although there was not a significant difference, the TSH levels in frailty group were slightly lower than those in non-frailty group (*p* =0.091). FT4 did not show any significant variation across three groups (*p* =0.310). When analyzing the TH sensitivity index in three groups, it was shown that TFQI(FT3) and FT3/FT4 reduced considerably in frailty group (*p <*0.001). However, TT4RI, TSHI and TFQI (FT4) did not show any significant differences compared to other groups (*p* =0.129, *p* =0.222, respectively).

### 3.4 Association of thyroid function and TH sensitivity index with sarcopenia

Out of the total number of 437 participants completed all assessments related to muscle, 184 individuals were diagnosed with frailty (**Table 2**). Similar to the general pattern, FT3, TFQI(FT3) and FT3/FT4 were significantly lower in the frailty group compared to non-frailty group (*p ≤*0.001). While the statistical analysis did not reveal a significant change in TSH (*p* =0.138), there was a noticeable downward trend.

**Table 2:** Association of thyroid function and TH sensitivity index with sarcopenia (n=437).

Variables	Non-sarcopenia (n=253)	Sarcopenia (n=184)	*p*-value
Age, years	79 (71~85)	87 (82~90)	<0.001
Female, %	112 (44.27)	74 (40.22)	0.398
FT3, pmol/L	3.95 (3.61~4.40)	3.77 (3.31~4.23)	<0.001
FT4, pmol/L	13.18 (12.35~14.26)	13.61 (12.69~14.68)	0.015
TSH, mIU/L	1.94 (1.34~2.87)	1.79 (1.04~2.73)	0.138
TSHI	2.42 (2.06~2.86)	2.45 (2.00~2.89)	0.743
TT4RI	26.11 (17.51~37.63)	24.52 (15.25~36.85)	0.344
TFQI(FT4)	-0.11 (-0.28~0.08)	-0.03 (-0.24~0.18)	0.041
TFQI(FT3)	0.02 (-0.15~0.25)	-0.04 (-0.29~0.15)	0.001
FT3/FT4	0.30 (0.27~0.33)	0.28 (0.24~0.31)	<0.001

Data are expressed as means ± standard deviations or medians (interquartile ranges) for continuous variables, and percentage (%) for categorical variables. P-value is calculated by U-test for continuous variables, Chi-square test for categorical variables.

TSHI, Thyroid Stimulating Hormone Index; TT4RI, Thyrotrophic Thyroxine Resistance Index; TFQI, Thyroid Feedback Quantile-based Index; FT3/FT4, Free Triiodothyronine/Free thyroxine.

### 3.5 The effect of thyroid parameters on frailty

A logistic regression analysis of the data was performed, and the association of thyroid function and TH sensitivity index with FRAIL scale scores was investigated by adjusting the multivariable-adjusted models (**Table 3**). The preliminary analysis (Model 1) revealed that FT3 (OR=0.436, 95%CI: 0.370-0.513, *p <*0.001), TSH (OR=0.943, 95%CI: 0.898-0.990, *p* =0.017), TSHI (OR=0.728, 95%CI: 0.632-0.838, *p <*0.001), TT4RI (OR=0.995, 95%CI: 0.991-0.999, *p* =0.007), TFQI(FT3) (OR=0.148, 95%CI: 0.102-0.214, *p <*0.001) and FT3/FT4 (OR=0.001, 95%CI: 0.000-0.004, *p <*0.001) exhibited negative correlations with the risk of frailty. However, no significant correlation was seen between FT4 (OR=0.996, 95%CI: 0.941-1.055, *p* =0.895), TFQI(FT4) (OR=0.694, 95%CI: 0.468-1.031, *p* =0.071) and frailty risk. Nevertheless, once demographic characteristics (gender and sex) were taken into account (Model 2) as well as the key influencing factors of frailty (Model 3), the previously observed association between TSH and frailty was no longer significant (OR=0.971, 95%CI: 0.924-1.021, *p* =0.255). After thoroughly accounting for secondary affecting factors, the strong negative correlation between FT3 (OR=0.640, 95%CI: 0.534-0.768, *p <*0.001), TH central sensitivity index, TSHI (OR=0.829, 95%CI: 0.708-0.971, *p* =0.020) and TFQI(FT3) (OR=0.358, 95%CI: 0.233-0.550, *p <*0.001), as well as TH peripheral sensitivity index FT3/FT4 (OR=0.006, 95%CI: 0.001-0.058, *p <*0.001), remained significant.

**Table 3:** Logistic regression analysis of the correlation of thyroid parameters and frailty risk.

OR (95%CI)	Model 1	Model 2	Model 3	Model 4
FT3	0.436* (0.370~0.513)	0.548* (0.463~0.650)	0.641* (0.535~0.768)	0.640* (0.534~0.768)
FT4	0.996 (0.941~1.055)	1.007 (0.950~1.067)	1.006 (0.940~1.077)	1.005 (0.937~1.076)
TSH	0.943* (0.898~0.990)	0.950* (0.904~0.998)	0.971 (0.924~1.021)	0.976 (0.929~1.025)
TSHI	0.728* (0.632~0.838)	0.747* (0.646~0.864)	0.818* (0.700~0.958)	0.829* (0.708~0.971)
TT4RI	0.995* (0.991~0.999)	0.995* (0.991~1.000)	0.997 (0.993~1.001)	0.997 (0.994~1.001)
TFQI(FT4)	0.694 (0.468~1.031)	0.731 (0.487~1.100)	0.826 (0.529~1.289)	0.827 (0.526~1.299)
TFQI(FT3)	0.148* (0.102~0.214)	0.230* (0.157~0.339)	0.348* (0.227~0.533)	0.358* (0.233~0.550)
FT3/FT4	0.001* (0.000~0.004)	0.008* (0.001~0.055)	0.006* (0.001~0.055)	0.006* (0.001~0.058)

Ordered Logistic regression was used to calculate the regression coefficient (OR) and 95% confidence interval (95%CI). *p-value <0.05 indicates a statistically significant difference.

Model 1, crude model; Model 2, adjusted for age and gender; Model 3, adjusted for all the factors adjusted in Model 2 plus the major influences on the development of the frailty, including BMI, nourishment state and exercise habits; Model 4, adjusted for all the factors adjusted in model 3 plus secondary influences on the development of the frailty, including drinking, hypertension, CHD, stroke and cognition.

TSHI, Thyroid Stimulating Hormone Index; TT4RI, Thyrotrophic Thyroxine Resistance Index; TFQI, Thyroid Feedback Quantile-based Index; FT3/FT4, Free Triiodothyronine/Free thyroxine.

## 4 Discussion

The results of this study indicated that in older individuals, there was a decline in serum FT3 and TSH levels as well as in TH sensitivity indicators [central sensitivity index of TH, comprising TFQI(FT3), and TH peripheral sensitivity index FT3/FT4] in those who were frail. Following the adjustment for several variables, the study found that changes in TSHI, TFQI(FT3) and FT3/FT4 were inversely correlated with the onset of frailty.

Thyroid hormones play a critical role in maintaining the health of the elderly, and they are closely related to the incidence of frailty (16). Our investigation found a gradual decline in serum FT3 and TSH levels as frailty levels increased. In order to confirm this pattern, additional studies were conducted on two specific groups: the oldest-old population and euthyroid patients. These analyses showed a comparable decrease in thyroid function in both groups. Patients with sarcopenia, a significant aspect of physical frailty, also experienced a comparable reduction in TH levels. The results showed that the presence of frailty in older individuals was linked to a steady decrease in serum FT3 and TSH. This phenomenon was not influenced by age or thyroid function state at the time of detection. Therefore, there was speculation that TH has function in both the occurrence and progression of frailty and may also serve as an indicator of individual levels of frailty to some degree.

Our findings were broadly consistent with, though not identical to, prior research. Guan et al. demonstrated that elevated TSH and reduced fT3 levels were both associated with frailty assessed by the FRAIL scale in the oldest-old community-dwelling adults (11). In contrast, Virgini et al. reported that subclinical hyperthyroidism (but not subclinical hypothyroidism) correlated with increased odds of frailty among elderly males (6). Notably, a separate study identified low TSH as an independent risk factor for frailty development (17). In this context, it should be noted that thyroid hormones and their feedback regulation systems can be influenced by numerous factors, rendering conventional measurements of FT3, FT4, and TSH potentially insufficient for comprehensive assessment of thyroid function (14). To address this limitation, recent researches have used composite calculation methods to measure thyroid hormone sensitivity and estimate thyroid homeostasis, following the proposal of acquired thyroid hormone resistance (18, 19). The TH sensitivity index utilized in this study consisted of three main components representing central sensitivity index of TH (TT4RI, TSHI, TFQI), and one component representing peripheral sensitivity index (FT3/FT4). Elevated TT4RI, TSHI and TFQI levels indicate reduced central sensitivity to TH, although a higher FT3/FT4 ratio suggests improved peripheral sensitivity. TT4RI and TSHI have contributed to identifying subclinical thyroid diseases, but their lack of clear reference ranges and susceptibility to outliers make them less reliable. The study utilizes TFQI in addition to TT4RI and TSHI to evaluate individual central sensitivity to TH, compensating for the limitations of the previous measures (13, 14). In addition, the FT3/FT4 ratio on behalf of deiodinase activity in peripheral blood, was considered to be the best indicator of the peripheral sensitivity of TH (20). Given the high prevalence of thyroid dysfunction in the elderly (21), this study examined a broad population first and conducted stratified analyses on individuals with normal thyroid function and octogenarians and beyond. Our study has found that the TH central sensitivity index, specifically TFQI(FT3), and peripheral sensitivity index FT3/FT4 of the frail elderly were lower compared to the non-frail elderly, even among the oldest-old population and those with adequate thyroid function. This indicated that frail elderly adults may have an increase in central sensitivity to TH and a decrease in peripheral sensitivity, suggesting thyroid hormone sensitivity alterations might operate independently of overt thyroid status. This reflected upstream hypothalamic-pituitary-thyroid (HPT) axis dysregulation rather than end-organ dysfunction. After accounting for other contributing factors of frailty, TFQI (FT3) and FT3/FT4 still exhibited a negative correlation with frailty, underscoring thyroid hormone sensitivity as a robust biomarker in frailty pathophysiology.

While the exact mechanism of how frailty affects thyroid hormone sensitivity remains unknown, this phenomenon may be explained by the well-known process of hormonal feedback control. The HPT axis is a crucial endocrine axis primarily responsible for the production and secretion of thyroid hormone (22). Under normal physiological conditions, TH enters hypothalamic and pituitary cells via specific transporters (e.g., MCT8, OATP1C1) (23). TH then regulates the hypothalamus to produce Thyrotropin-Releasing Hormone (TRH), which stimulates the pituitary to synthesize and secrete TSH. TSH subsequently binds to its receptor (TSHR) on the thyroid gland, inducing TH production (24). This completes a classic negative feedback loop that maintains physiological levels of TSH and TH within the organism. However, our findings indicated that frail elderly patients exhibited concurrently low serum levels of both FT3 and TSH. This reflected a failure of the hypothalamus and pituitary to mount the expected compensatory TSH increase in response to low FT3 levels—a paradoxical finding. This may be attributed to specific alterations in hypothalamic-pituitary neurons in frailty, impairing their normal sensitivity to TH (22, 25). Future studies elucidating this mechanism could thereby pave the way for breakthrough therapeutic frameworks in frailty management. Deiodinases play a vital role in mediating the biological effects of TH on peripheral target tissues. These enzymes convert T4 into the biologically active T3, thereby increasing local T3 concentrations and enhancing target tissue sensitivity (26). With aging, impaired deiodinase activity reduces this conversion efficiency, leading to decreased T3 generation. This subsequently disrupts T3-dependent energy metabolism and muscle regulatory pathways, accelerating muscle wasting (22). These mechanisms explain why age-related frailty correlates with reduced FT3/FT4 ratios (20), which was consistent with our findings.

This study investigation confirmed that the patterns of thyroid hormone and its sensitivity in frail population would change as frailty progresses. It was clear that evaluating only thyroid hormone levels could be insufficient to properly understand thyroid homeostasis in frail older adults. The application of thyroid hormone sensitivity indices, particularly TFQI (FT3) and FT3/FT4, may effectively overcome these constraints, thus highlighting thyroid hormone sensitivity as a pivotal biomarker in the pathophysiology of frailty to assess individual’s frailty and demonstrating its clinical utility for assessing geriatric frailty in older adults. This study focused on the correlation between thyroid hormone sensitivity and frailty, a subject that has not been investigated in either local or international literature. However, there were still certain constraints and limitations. Initially, as this was a cross-sectional study, no causal inferences can be made. Pertinent topics are being pursued to further substantiate these findings. Furthermore, the responses about medical history were highly subjective, which introduces the possibility of bias in our findings. Other factors concerning the development of frailty, like hereditary characteristics, socioeconomic position and inappropriate drug usage, have not been evaluated.

## 5 Conclusion

The thyroid function in elderly individuals with frailty exhibited the following characteristics: the levels of serum FT3 and TSH decreased in frail patients, while there was no alteration in serum FT4. Out of all the variables, FT3 has the highest correlation with frailty. Frailty, as measured by the FRAIL scale, was found to be related to a decrease in both the central sensitivity index of thyroid hormone [TSHI and TFQI(FT3)] and the peripheral sensitivity index (FT3/FT4). Thyroid function and thyroid hormone sensitivity indices, particularly FT3, TFQI(FT3) and FT3/FT4, can serve as conveniently accessible indicators that are essential for assessing frailty.

## Data Availability

The original contributions presented in the study are included in the article/supplementary material. Further inquiries can be directed to the corresponding authors.
